# Metainflammation and autonomic instability in the genesis of anxiety

**DOI:** 10.3389/fendo.2026.2008588

**Published:** 2026-10-29

**Authors:** Milica M. Borovcanin, Branislav Milovanovic, Nikola Markovic, Ivica Petrovic

**Affiliations:** 1 Department of Psychiatry, Faculty of Medical Sciences, University of Kragujevac, Kragujevac, Serbia; 2 Psychiatric Clinic, University Clinical Centre Kragujevac, Kragujevac, Serbia; 3 National Institute for Heart and Blood Vessels “Dedinje”, Belgrade, Serbia; 4 School of Medicine, University of Belgrade, Belgrade, Serbia; 5 Department of Pathophysiology, Faculty of Medical Sciences, University of Kragujevac, Kragujevac, Serbia

**Keywords:** anxiety, autonomic instability, immunometabolism, metainflammation, neuroinflammation

## Abstract

Anxiety is a complex condition that encompasses a range of mental disorders. It can manifest as a core symptom in various neurotic disorders, including anticipatory anxiety, pervasive anxiety, panic attacks, obsessive-compulsive, conversion and somatoform disorders. Anxiety is often linked to autonomic instability, which can be both observed and felt subjectively. However, anxiety does not arise solely from mental disorders; it can also occur alongside somatic conditions related to autonomic instability and cardiorenometabolic syndrome. Obesity and cardiorenometabolic syndrome, which can lead to diabetes and are associated with metainflammation, represent significant societal challenges that adversely impact mental functioning. In this context, neuroinflammation should be viewed as a consequence within the central nervous system of systemic inflammation and may be related to anxiety. Anti-inflammatory strategies have been shown to stabilize mental functioning, exhibit anxiolytic properties, and promote balance in the autonomic nervous system and metabolic homeostasis, with interconnected effects. While certain medications and procedures can influence these interrelated processes, they may also lead to undesirable side effects. This narrative review aims, for the first time, to jointly explore the triad of metainflammation, autonomic instability, and anxiety, in an attempt to clarify these important associations for future research and implementation in clinical practice.

## 1 Introduction

Signs of autonomic nervous system imbalance should not be interpreted from a single perspective in the modern neuroscientific era. There are no simplified cases in clinical practice, especially given the potential impact of infection on multiple organ systems functioning. Complex cascades are involved, and autonomic instability can arise through inflammatory and metabolic pathways. This narrative review aimed to highlight the need for a holistic approach and the potential of targeting inflammation and vagus activity as preventive and therapeutic strategies in cardiorenometabolic syndrome and type 2 Diabetes Mellitus (DM), with simultaneous amelioration of the resulting anxiety.

From a psychiatric perspective, this vegetative symptomatology is frequently observed and closely linked with inner perception and feelings. Anxiety is often associated with neurovegetative symptoms, which can be observed or felt subjectively. However, it is not specific to a single mental disorder. Anxiety is primarily related to exacerbation, functioning as an alarm mechanism in threatening situations, but it can also be prolonged, causing severe mental and somatic imbalance. It is a core symptom of different neurotic disorders and can also be a constituent of depression, psychosis, delirium, dementia, addiction, personality disorders, etc. Genuine anxiety is especially important in this context, as it can appear in various neurotic disorders as the dominant symptomatology, including anticipatory anxiety, pervasive anxiety, panic attacks, obsessive-compulsive disorder, conversion disorders, and somatoform disorders. Within our scope are specifically acute stress reaction and post-traumatic stress disorder, panic disorder, and numerous possible conversion reactions, which may be the most representative in the group of neurotic and stress-related disorders. Understanding how individuals react to stress and exploring coping strategies is fascinating, particularly in the context of preventive strategies for somatic morbidity (1).

It is known that *de novo* diabetes could be related to the identification of acute stress (2). In contrast, chronic and prolonged stress is commonly linked to changes in immunometabolism and the possible onset of cardiorenometabolic syndrome and DM. DM is recognized as a leading cause of autonomic neuropathy (3, 4). A distinct neural baroreflex pathway has been identified in cardiometabolic syndrome, establishing a connection to autonomic dysfunction (5). Hyperarousal, somatoform disorders, and conversion disorders, although initially psychologically induced, can impose biochemical and physiological burdens that ultimately result in new pathophysiological states and somatic illnesses. This progression illustrates a “tipping point” phenomenon. Anxiety, moreover, does not arise solely from mental disorders; it can also occur alongside somatic conditions related to autonomic instability and cardiorenometabolic syndrome and could involve underlying inflammation. An integrated discussion is required to examine the interplay of multiple factors contributing to the genesis of anxiety and to identify a balanced approach for addressing this psychopathology.

Among other aspects, the neurobiology of anxiety is related to neuroinflammation in the brain as a substrate. Neuroinflammation cannot be detected by conventional methods and is not assessed in routine clinical practice. Standard practice typically involves promptly excluding neurological problems with conventional neuroimaging and referring patients with behavioral changes to a psychiatrist. More in-depth and sophisticated neurobiological and neurochemical analyses are generally lacking. In this narrative review, we repeatedly examine the causes of neuroinflammation in anxiety. As one piece of the puzzle, metainflammation in the periphery could be linked to neuroinflammation through vagal innervation (6). We aim to integrate the following components into a bidirectional cascade: the impact of stress, activation of the Hypothalamic-Pituitary-Adrenal (HPA) axis and cortisol secretion, Autonomic Nervous System (ANS) function, and immunometabolic disturbances, culminating in neuroinflammation associated with anxiety.

## 2 Acute and chronic stress leading to anxiety

Without fear, life would be diminished, and our behavior would be completely different (7). A moderate level of fear can be beneficial for concentration, for showcasing our knowledge and skills, and for achieving optimal results in specific situations. On a biological level, fear drives us to take action to protect ourselves from danger and supports our survival. In a threatening situation, the reaction could be explained globally by the famous tree F: fight (resistance), flight (adaptive coping), or freeze (maladaptive coping). Typical behaviors that express fear include running away, avoiding situations, seeking refuge in safe places, moving cautiously, frequently looking behind, being vigilant, experiencing restlessness, etc. Fear can manifest physically through symptoms such as heart palpitations, shortness of breath, sweating, trembling of the hands or other parts of the body, fainting, muscle tension, and fluttering in the stomach or other somatic sensations (8). All the somatic symptoms listed should not be immediately labelled as psychologically related problems; but first, it is necessary to exclude possible somatic causes.

We must keep in mind that fear is a reaction to a specific, identifiable external threat. The range of stressors involved is wide and includes psychosocial stressors, various physical traumas, infectious agents, intoxication, radiation, etc. In contrast, Freud described anxiety as an unclear, internal threat that arises from unresolved conflicts. Adaptive anxiety serves as a warning, urging individuals to take action to prepare for potential danger. If the Ego does not function properly for an extended period, the individual may develop chronic, debilitating anxiety.

Further, panic disorder involves recurrent, unpredictable episodes of severe anxiety, known as panic attacks, that are not confined to specific situations. Sudden onset of palpitations, chest pain, feelings of suffocation, fainting, and sensations of unreality, including depersonalization and derealization, are common symptoms (9). There is often a secondary fear associated with death, which can include concerns about losing control or descending into madness. A person experiencing a panic attack often feels overwhelming fear and intense physical symptoms, and it is often accompanied by anticipatory fear of having another attack. The described clinical presentations can resemble those seen in, for example, epileptic seizures, a heart attack, or hypoglycemia, making it urgently necessary to distinguish between these states.

## 3 Role of amygdala in stress and immunometabolism

The amygdala plays an important role in understanding the etiopathogenesis of stress. It is crucial in generating stress, while the hippocampus and hypothalamus are important for the cognitive aspects of recognizing experiences and recalling memories. The amygdala is the brain region responsible for both acquiring and expressing fear (7, 10). Located in the medial temporal lobe, the amygdala consists of 13 nuclei, three of which are involved in the fear response: the basal amygdala, the lateral amygdala, and the central nucleus. Stimuli received by the thalamus are sent to the lateral amygdala and then to the central nucleus, forming the “short loop” pathway. The basal amygdala links the lateral amygdala with the central nucleus. Signals are transmitted to the lateral amygdala through the “long loop” pathway originating from the sensory cortex, insula, and prefrontal cortex. This information is projected to the brainstem and hypothalamus, resulting in autonomic and behavioral manifestations of an acute attack of fear. The lateral amygdala is a brain region that plays a crucial role in memory consolidation and the plasticity associated with fear conditioning.

The balance of excitatory and inhibitory neurotransmission is disturbed in acute inflammation, leading to heightened amygdala reactivity and impaired regulatory functions of the prefrontal cortex (11). Elevated serum levels of proinflammatory cytokines have been measured in subjects with anxiety disorders and reflect the severity of symptoms (12). As summarized by Cătălina et al. (13), Interleukin-6 (IL-6) activates the amygdala and hypothalamus, resulting in hypervigilance and somatic anxiety symptoms, while an attenuated diurnal IL-6 rhythm is associated with amygdala hyporeactivity and depressive symptoms (14). Tumor Necrosis Factor-alpha (TNF-α) acts on the HPA axis and exacerbates the physiological and psychological symptoms of anxiety (15).

Chronic inflammation can alter neurotransmission, particularly in pathways involving serotonin and Gamma-Amino Butyric Acid (GABA). These mechanisms may play a role in generating anxiety. In chronic inflammation, neural remodeling of the hippocampus contributes to impairments in stress adaptation and memory consolidation that are critical in the pathogenesis of anxiety disorders (16). Chronic anxiety-induced stress is characterized by increased sympathetic activity and reduced parasympathetic tone, which disrupt homeostatic mechanisms within internal organs (17).

In addition to autonomic and behavioral manifestations, higher baseline metabolic Activity in the Amygdala (AmygA) predicted new-onset DM regardless of other risk factors (18). Synergistically, higher adiposity increased DM risk only in the presence of higher AmygA. Higher AmygA was associated with greater insulin resistance.

## 4 Chronic stress and immunometabolism

Bridging neuropsychiatric and internal diseases, systemic inflammation is characterized by elevated levels of IL-6, TNF-α and C-Reactive Protein (CRP), and can be further perpetuated by chronic activation of the HPA axis and dysregulation of the ANS, creating a vicious cycle of disease progression (13) (**Figure 1A**). Salivary cortisol and alpha-amylase have been identified as principal markers of the acute stress response, and IL-6, TNF-α and CRP indicate chronic inflammation when emotional distress is prolonged (19).

**Figure 1:**
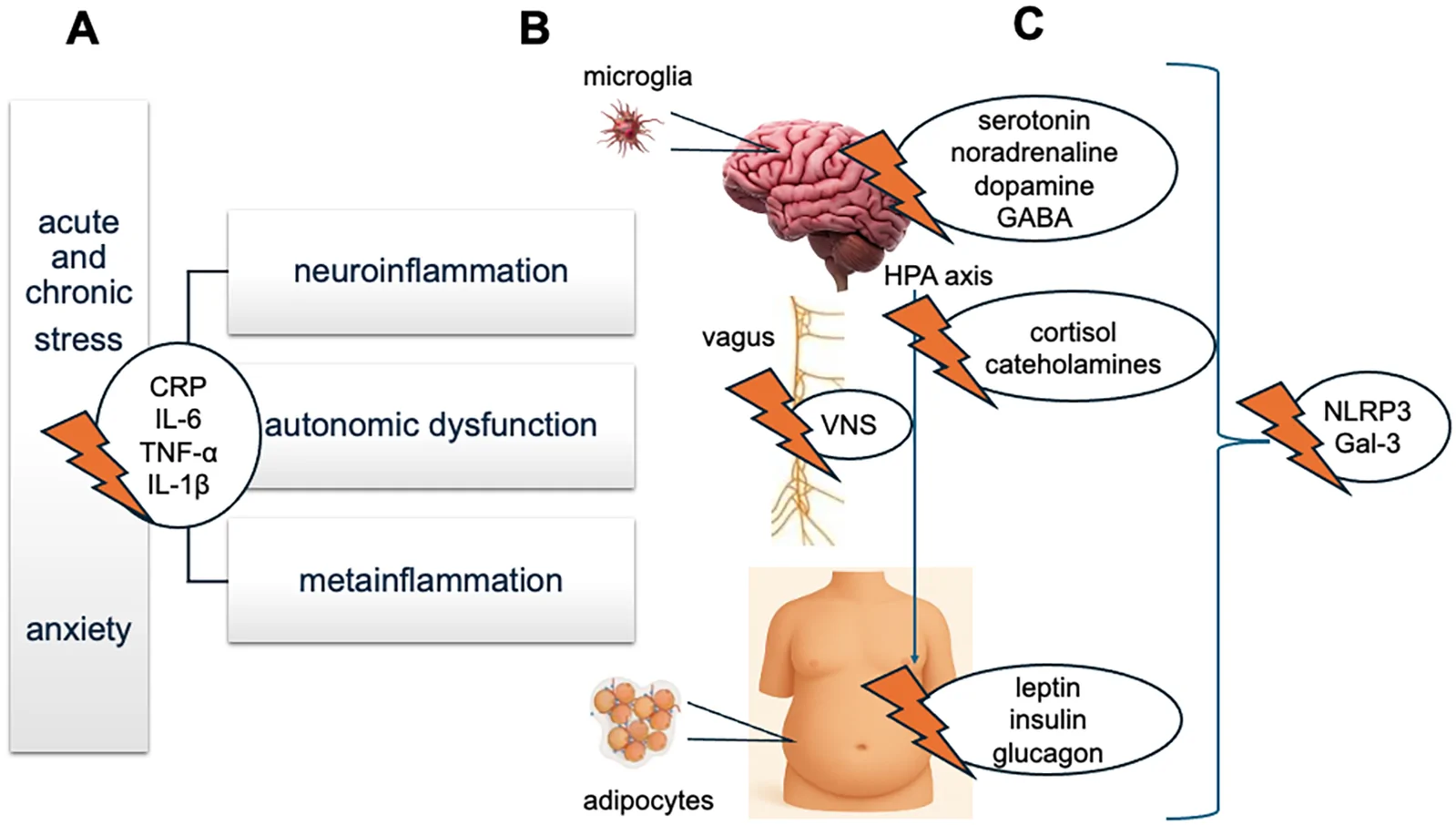
Legend. Key strategies for the simultaneous resolution of neuroinflammation, metainflammation, and autonomic dysfunction leading to anxiety. **(A)** Acute and chronic stressors may induce both neuroinflammation and metainflammation, potentially resulting in autonomic instability. Conversely, autonomic disturbances may also contribute to these inflammatory processes, collectively generating anxiety. **(B)** Neuroinflammation involves activated microglia, whereas metainflammation is observed in adipocytes. Proinflammatory biomarkers implicated in both conditions include C-reactive protein (CRP), Interleukin-6 (IL-6), Tumor Necrosis Factor-alpha (TNF-α), and Interleukin-1 beta (IL-1β). **(C)** Established pharmacological agents targeting neurotransmitters (serotonin, noradrenaline, dopamine, GABA, etc.) and incretins (leptin, insulin, and glucagon) may serve as anti-inflammatory interventions while also addressing autonomic and metabolic dysfunction. The Hypothalamic-Pituitary-Adrenal (HPA) axis, involving cortisol and catecholamines, is another potential therapeutic target. Vagal Nerve Stimulation (VNS), as a specific procedure, could be beneficial for these purposes. Emerging therapeutic targets include molecules present in both peripheral tissues and the brain, such as NOD-like receptor protein 3 (NLRP3) and Galectin-3 (Gal-3).

Constant chronic stress with elevated cortisol enhances the stimulatory effect of neuropeptide Y, increases hunger, and is associated with a positive caloric balance and the development of obesity (20). Over time, a positive energy balance leads to the development of two similar but distinct, interconnected types of obesity: hypertrophic and hyperplastic. One type of adaptation is always dominant, depending on the capacity for adipogenesis, the amount and localization of adipose tissue, genetics, gender, age, and metabolic context (21).

The hyperplastic type of obesity is generally defined by more functional adipocytes, lower metabolic inflammation, less pronounced hyperinsulinemia, better insulin sensitivity of peripheral tissues, and relatively preserved metabolic leptin signaling (22, 23). Hypertrophic adipocytes exhibit metabolic dysfunction, which leads to impaired adipokine secretion and increased inflammatory signaling. These changes contribute to the development of metabolic inflammation and insulin resistance in both adipose tissue and other peripheral tissues (24, 25). This type of obesity is characterized by a higher degree of insulin resistance, manifested by compensatory hyperinsulinemia, increased leptin secretion compared with the hyperplastic type, the onset of leptin resistance, a weaker anorexigenic signal, and additional appetite disturbance (26, 27).

Thus, it seems that the genetic and epigenetic capacity for adipocyte hyperplasia determines the level of hypoxia in adipose tissue and, consequently, the degree of impaired insulin sensitivity, the extent of metabolic inflammation, and the level of leptin secretion and leptin resistance (28). In summary, the most severe form of the disorder is associated with the hypertrophic type, due to simultaneous pronounced hyperinsulinemia and hyperleptinemia, which ultimately leads to disruption of the central interpretation of the peripheral energy signal and the sensation of satiety, resulting in even greater obesity and the establishment of a vicious cycle (29).

## 5 Communication between adipose tissue and brain *via* autonomic nervous system

Communication between adipose tissue and the CNS is bidirectional, and *via* the ANS it can affect the metabolism of adipose tissue. Through its ascending fibers, the vagus nerve conveys information about meal size and duration, while through its descending fibers it controls the processes of food digestion, absorption, and permeability at the epithelial level (30–32). Experimental and mechanistic evidence indicates that obesity-associated hyperleptinemia can induce leptin resistance in vagal afferent neurons, with a consequent disruption in the interpretation of the energy signal in the CNS during a meal (33). Sympathetic activity, on the other hand, exerts its effects on adipose tissue via β 2 adrenergic receptors and regulates energy expenditure. In chronic states of stress, sympathetic activity is most often increased, but the problem arises when, due to chronic stimulation, downregulation of β 2 adrenergic receptors occurs, leading to a blunted catecholamine-induced lipolytic response (34).

In ascending communication, the intestinal endocrine L cells play a significant role in the production and release of Glucagon-Like Peptides, GLP-1 and GLP-2. These hormones, released from the gastrointestinal tract in response to nutrients and called incretins, are very important for the postprandial response and the secretion of insulin and glucagon, the feeling of hunger, and gastric motility, as well as maintaining the integrity and permeability of the intestinal mucosa (35, 36). Patients with obesity-related diseases often experience impaired postprandial hormone secretion. This results from chronic overeating, which overloads nutrient sensors on L cells and leads to their functional desensitization (37). At later stages of the continuum, disturbed vagal activity may further affect incretin secretion, intestinal barrier permeability, endotoxemia development, and metabolic inflammation (38). Gut motility may be disturbed because of ANS dysregulation, and gut microbiota composition may be altered by stress and implicated in gastrointestinal inflammation and anxiety (39).

## 6 Metainflammation inducing neuroinflammation and anxiety by vagal activation

The brain’s regulation of the organism’s functioning is often seen as the primary mechanism, yet it also responds in the opposite direction. Communication from the periphery occurs through the Blood-Brain Barrier (BBB), deep lymphatic nodes, and the vagus nerve, indicating that the immune system, ANS and metabolism act simultaneously on central regulation and behavior, and none of them can be disregarded. Positive affectivity and resilience are linked to epigenetic modifications of genes involved in synaptic plasticity and in the endocrine, immune, and vascular systems, for example, at the glucocorticoid receptor, the IL-6 gene, or in epigenetic adaptations of the BBB (40). Disturbance of this pathway is associated with pathological conditions and may serve as a target for innovative combined therapeutic strategies.

Debate continues: is inflammation a cause or a consequence? Anxiety can clearly affect immune defense and cause immune disturbance, but it can also result from inflammation caused by clearly diagnosed somatic diseases (41). The concept of sterile inflammation (42) highlights these changes and emphasizes not only viruses, bacteria, or other microorganisms, but also the possible impact of various toxins, nutritional factors and dietary habits, disturbed circadian rhythm, mental hygiene, psychotherapy, etc.

Metabolic inflammation, or metainflammation, is a state of low-grade inflammation caused by excessive nutrition. At the core of this chronic process is an increase in adipocyte volume associated with adipozopathy, adipocyte decoy, and local inflammation (**Figure 1B**). In the long term, metabolic inflammation further deteriorates adipocyte function, increases local inflammation, and worsens insulin resistance, leading to the development of lipid and glucose regulation disorders. Unlike pronounced acute inflammation or infection, this condition very often remains unrecognized because most of the parameters used clinically for its detection are within the normal range or only slightly elevated. The findings prompted the integration of multiple perspectives and markers associated with chronic metabolic inflammation, leading to the development of the metaLGIscore (43). This parameter is widely accessible and demonstrates superior specificity and sensitivity compared to commonly used markers across the spectrum of obesity-related diseases.

Neuroinflammation results from a series of immunological events triggered by infection, autoimmunity, alarmins, brain injury, or metabolic disturbances (**Figure 1B**). For example, IL-1β penetrates the BBB and activates microglial cells, causing neuroinflammation, disrupting neurotransmission, potentiating amygdala reactivity, and impairing the regulatory functions of the prefrontal cortex, together provoking anxiety (44). An animal model of sepsis confirmed the resulting neuroinflammatory changes as well as long-term persistent anxiety (45). Classical neuroinflammation is seen in infectious or autoimmune encephalitis and could be followed by agitation or anxiety as a sequela (46). Autoimmune processes could be focused on the autonomic nervous system (47). In Multiple Sclerosis (MS), a neuroinflammatory and neurodegenerative disease that can involve anxiety, we have explored the personality traits and coping strategies on quality of life (48), and a positive correlation was established between overall autonomic dysfunction and IL-6 serum levels (49). Lower-grade neuroinflammation, localized perivascularly, intensifying with a type 2 immune response in exacerbation and linked with anxiety, is seen in schizophrenia (50, 51). There is a tendency to correlate parameters reflecting neuroinflammation and systemic inflammation with clinical, accessible, and affordable measurements (52).

Post-infective syndromes can be accompanied by autonomic instability and fatigue, as well as anxiety. For example, Postural Orthostatic Tachycardia Syndrome (POTS), which is related to syncope, has been associated with baroreceptor dysfunction and sympathetic predominance. In contrast, Chronic Fatigue Syndrome (CFS)-related POTS is characterized by parasympathetic impairment and impaired short-term baroreflex regulation (53). One example is infection with *Coxiella burnetii*, which can induce autonomic dysfunction with syncope that persists and contributes to the development of CFS or Myalgic Encephalomyelitis (ME) (54). Acute infections, as well as the consequences of past infections, may be disregarded, and their impact on autonomic dysfunction and the onset of anxiety may be underestimated. Poor handling of syncope as a threat to personal integrity, humiliation, or even fear of death can lead to an unconscious suppression of tension in individuals, which may later manifest as panic attacks or agoraphobia, and may even be complicated by depression.

Further, evidence points to the possibility of Severe Acute Respiratory Syndrome CoronaVirus 2 (SARS-CoV-2)-induced diabetes (55). In such cases, diabetes onset is associated with host characteristics and social determinants. However, Coronavirus Disease 2019 (COVID-19) may also directly and indirectly influence pancreatic β-cell function and insulin sensitivity through mechanisms such as infection, treatment, potential autoimmunity, and persistent viral presence in multiple organs. These include adipose tissue, endothelial dysfunction, and a sustained hyperinflammatory state. It has even been discussed that this variety of diabetes should be classified as a post-COVID syndrome, in addition to traditional classifications of diabetes (e.g., type 1 or type 2), considering its pathophysiology and future management.

Clinical evidence indicates that autonomic dysfunction is more pronounced in patients with DM and severe aortic stenosis, with a predominant impact on parasympathetic modulation (56). Glucose dysregulation, including glycemic variability, reactive hypoglycemia, and postprandial spikes, may trigger systemic inflammation and temporary autoantibody production, autonomic dysfunction and anxiety (57). For example, sudden cardiac death, which is most threatening in patients with schizophrenia, could be related to all of these mechanisms: excessive anxiety, systemic and neuroinflammation, autonomic dysfunction, and akathisia, within cardiorenometabolic syndrome that could be drug-related (51, 58).

Valuable information has been proposed after the analysis of retrospective and prospective studies, showing that patients with depression or anxiety have an elevated risk for lung, hepatobiliary-pancreatic, hematological, and kidney, ureter, and bladder cancers (55). In some cases, anxiety can be observed even in the prodrome as a nonspecific symptom, for example, in pancreatic cancer (59). Anxiety may be added as a clinical marker, along with observed unintended weight loss and new-onset DM, as a reminder to consider pancreatic cancer in these patients (60).

Painful experiences most frequently seen in oncological problems are linked to both acute and chronic stress, as well as acute and chronic inflammation (61). Acute stress from intensive neuropathic pain is related to acute inflammation and marked behavioral changes called “sickness behavior”, whereas chronic prolonged pain is associated with chronic inflammation, fatigue, and anxiety. If amygdala reactivity is heightened, it could amplify visceral pain perception (62). Reduced vagal tone may increase sympathetic dominance, impair regulation of inflammatory responses, and increase susceptibility to fatigue, anxiety, depressive symptoms, sleep disturbances, and pain. Decreased Heart Rate Variability (HRV) in breast cancer patients has been associated with increased inflammatory activity and anxiety (63). HRV is a comprehensive autonomic-immune-affective phenotype rather than a purely cardiac signal and is considered a candidate marker of regulatory state (64).

## 7 Further directions

When we started designing this manuscript, we faced the challenge of presenting all the new concepts together. To the best of our knowledge, no work in the popular medical databases has considered neuroinflammation, the new concept of metainflammation, and autonomic dysfunction together and discussed them jointly in relation to anxiety. We therefore abandoned the idea that this manuscript needed to be a systematic review and instead presented it as a narrative mini-review, placing it at the border with a perspective paper and making it more attractive to readers in different fields.

This clinical and research vignette on the triad of anxiety, metainflammation, and autonomic instability has as its major aim the emphasis on the need for an integrative approach. Precision medicine and robust data analysis must generate new clinical guidelines for prevention, early recognition and therapy.

Firstly, it must be emphasized that psychotherapy balances ANS tone, ameliorates inflammation, and stabilizes metabolic functioning (13). It is well known that the simultaneous psychopharmacological treatment of somatic disease could lead to better outcomes (65). In clinical practice, it is necessary to avoid polypharmacy and minimize undesirable drug interactions. Therefore, the ideal goal is to use a polyvalent drug that has anti-inflammatory properties while preserving metabolism and balancing the autonomic nervous system (summarized in **Figure 1**).

Current medications for treating anxiety symptoms and comorbid stress-related disorders are based on correcting neurotransmitter imbalances of serotonin, dopamine, noradrenaline, etc. (**Figure 1C**). However, these drugs have broader effects on the immune system, metabolism, and ANS, and exploring their repurposing potential is a current hot topic (66). Beta-blockers are useful for addressing cardiological symptomatology and ANS dysregulation, and they are also included in the protocol for treating acute stress reactions (67). Sodium-Glucose Cotransporter 2 (SGLT2) inhibitors have been explored for their impact on the ANS by reducing sympathetic tone in various cardiovascular and metabolic disease states, but results remain conflicting (68, 69). In the hypothesis and theory article, Das (70) discussed how acting on lipids seems to have simultaneous beneficial effects on cardiorenometabolic syndrome, inflammation, ANS, and anxiety.

Vagus Nerve Stimulation (VNS) could alleviate anxiety in long COVID symptoms (**Figure 1C**). The positive aspects of this VNS procedure were explained through the parasympathetic-sympathetic reflex arc in relation to pulmonary problems. Improvements in various aspects of mental functioning, including anxiety, were linked to reductions in systemic inflammation, enhanced cerebral blood flow, neurotransmitter modulation, and restoration of adequate autonomic function, also triggering the HPA axis (71).

Unwanted outcomes of prescribing various drugs include the induction of anxiety. Patients with obesity were treated with tirzepatide, and the most frequent complications were dyslipidemia, hypertension and anxiety (72). The results also underscore the importance of maintaining clinical vigilance for anxiety symptoms after discontinuation of GLP-1RA therapy, possibly due to its wide spectrum of action (73). These phenomena should be considered in the context of addictions and reward-punishment circuits.

Targeting the NOD-Like Receptor Protein 3 (NLRP3) inflammasome pathway, an intracellular sensor detecting microbial motifs, could in the future be established as a new polyvalent protocol associated with neuroinflammation, immunometabolism and autonomic instability in psychiatric disorders (74–78) (**Figure 1C**). Galectin-3, a member of the lectin family, could also be considered useful and has already been discussed for these purposes (79).

## 8 Concluding remarks

This is primarily an attempt to achieve a clinically balanced approach. Limitations of this mini-review could include its broad scope, its transdisciplinary and translational approach, and potential bias stemming from the authors’ narrow fields of interest. There is a risk of focusing mainly on psychological aspects and the psychological origins of anxiety. Somatic components and causal treatment in this case could be irrationally disregarded. The key aspect of a diagnostic algorithm is first to rule out any physical causes.

However, excessive and continuous exploration of the patient and invasive diagnostics could reinforce the patient’s belief that there is a clear organ dysfunction that must be identified and treated. Both scenarios are therefore possible: firstly, when somatization predominates, and secondly, when anxiety is a consequence of somatic illness. The predominant formatting factor should be identified in every case.

In this context, autonomic instability could be considered as an alarm that is activated in these disturbances. In acute stress, these mechanisms may be excessively activated, while in post-traumatic stress disorder and chronic stress, they are continuously overactivated. Vagal activation and inflammatory phenomena may occur in parallel and can also mutually generate or potentiate each other. Metainflammation communicated through the brain-fat and brain-gut axes can induce neuroinflammation *via* the vagus nerve, contributing to anxiety.

Doctors should focus on the whole person, not just one organ or disease. The crucial aspect is the networking within the organism, intertwining the nervous, immune, and vascular systems. It also includes interactions with adipose tissue and the digestive system, all of which communicate through neurotransmitters. It may seem complicated, but a good anamnesis and longitudinal approach could delineate the formative factors and major therapeutic targets. Big data analyses in precision medicine will integrate all this knowledge for the benefit of patients.
